# HLA-B27 and Human β2-Microglobulin Affect the Gut Microbiota of Transgenic Rats

**DOI:** 10.1371/journal.pone.0105684

**Published:** 2014-08-20

**Authors:** Phoebe Lin, Mary Bach, Mark Asquith, Aaron Y. Lee, Lakshmi Akileswaran, Patrick Stauffer, Sean Davin, Yuzhen Pan, Eric D. Cambronne, Martha Dorris, Justine W. Debelius, Christian L. Lauber, Gail Ackermann, Yoshiki V. Baeza, Tejpal Gill, Rob Knight, Robert A. Colbert, Joel D. Taurog, Russell N. Van Gelder, James T. Rosenbaum

**Affiliations:** 1 Casey Eye Institute, Oregon Health & Science University, Portland, Oregon, United States of America; 2 Division of Rheumatology, Oregon Health & Science University, Portland, Oregon, United States of America; 3 Division of Rheumatology, University of Washington, VA Medical Center, Seattle, Washington, United States of America; 4 Moorfield's Eye Institute of London, London, United Kingdom; 5 Department of Ophthalmology, University of Washington, Seattle, Washington, United States of America; 6 Department of Rheumatology, University of Texas Southwestern, Dallas, Texas, United States of America; 7 University of Colorado Boulder, Boulder, Colorado, United States of America; 8 Dever's Eye Institute, Portland, Oregon, United States of America; 9 Department of Molecular Microbiology & Immunology, Oregon Health & Science University, Portland, Oregon, United States of America; 10 Pediatric Translational Research Branch, National Institute of Arthritis, Musculoskeletal and Skin Diseases, National Institutes of Health, Baltimore, Maryland, United States of America; 11 Howard Hughes Medical Institute, University of Colorado Boulder, Boulder, Colorado, United States of America; Charité-University Medicine Berlin, Germany

## Abstract

The HLA-B27 gene is a major risk factor for clinical diseases including ankylosing spondylitis, acute anterior uveitis, reactive arthritis, and psoriatic arthritis, but its mechanism of risk enhancement is not completely understood. The gut microbiome has recently been shown to influence several HLA-linked diseases. However, the role of HLA-B27 in shaping the gut microbiome has not been previously investigated. In this study, we characterize the differences in the gut microbiota mediated by the presence of the HLA-B27 gene. We identified differences in the cecal microbiota of Lewis rats transgenic for HLA-B27 and human β2-microglobulin (hβ2m), compared with wild-type Lewis rats, using biome representational in situ karyotyping (BRISK) and 16S rRNA gene sequencing. 16S sequencing revealed significant differences between transgenic animals and wild type animals by principal coordinates analysis. Further analysis of the data set revealed an increase in *Prevotella spp.* and a decrease in Rikenellaceae relative abundance in the transgenic animals compared to the wild type animals. By BRISK analysis, species-specific differences included an increase in *Bacteroides vulgatus* abundance in HLA-B27/hβ2m and hβ2m compared to wild type rats. The finding that HLA-B27 is associated with altered cecal microbiota has not been shown before and can potentially provide a better understanding of the clinical diseases associated with this gene.

## Introduction

The role of the gut microbiota in diseased and healthy states has become increasingly apparent, as evidenced by its importance in a number of conditions including metabolic syndrome, type 1 diabetes, multiple sclerosis, rheumatoid arthritis, and inflammatory bowel disease [1]–[7]. Many of these diseases are also influenced by the genes of the major histocompatibility complex (MHC), otherwise known as human leukocyte antigens (HLA) in humans. In many conditions, the HLA alleles confer greater disease risk than other genetic factors identified by genome wide association studies [8]. Ankylosing spondylitis (AS), a potentially disabling condition that results in axial and sometimes peripheral arthritis is highly associated with an MHC class I molecule, HLA-B27, with ∼ 90% of patients with AS carrying the HLA-B27 allele. The mechanism by which HLA-B27 predisposes to disease is not well understood, although several hypotheses have been postulated [9]–[11]. Our study tests the hypothesis that HLA-B27 alters the repertoire of the gut microbiome [11], which may be one mechanism by which it predisposes to disease.

Studies in both humans and rodents support this hypothesis [12]. Bacteria including *Shigella, Salmonella, Yersinia,* and *Cambpylobacter* are well-characterized triggers for HLA-B27-associated reactive arthritis [13]–[16]. Cross reactivity between monoclonal antibodies to HLA B27 and Gram-negative bacteria has been reported [14], [17], [18]. There is also sequence homology between a nitrogenase from *Klebsiella* and HLA-B27, although the significance of this finding has been debated [13], [19], [20]. Raising HLA-B27 transgenic rats in a germ-free environment prevented both gut and joint inflammation that otherwise spontaneously occurs in these rats [21]. Subsequent studies showed that mono-association of HLA-B27 transgenic rats with *Bacteroides vulgatus* in a germ-free environment was sufficient to re-establish colitis in these animals [22], [23]. A series of studies by Granfors et al have demonstrated marked effects of HLA-B27 on innate immunity and host defense *in vitro*, including effects on bacterial invasion of cells, intracellular persistence, intracellular signaling, and cytokine production [24]–[28]. Penttinen et al. showed that HLA-B27-transfected monocytes had enhanced inflammatory responses to lipopolysaccharide found on bacterial cell walls [26], [29]. Moreover, disease-prone HLA-B27 transgenic rats exhibit a variety of dendritic cells abnormalities [30], [31] including disrupted trafficking of dendritic cells from the gut to the mesenteric lymph nodes [32]. It is plausible that a dysregulated immune response in HLA-B27 individuals alters the composition of the gut microbiome, and that in turn, the altered gut microbiota contributes to disease pathogenesis.

There are various types of animal models of HLA-B27 disease. The HLA-B27 transgenic rats on a Lewis background created by crossing the 21–3 and 283–2 strains express high copy numbers of the HLA-B27 subtype B27:05 and human beta 2 microglobulin (hβ2m) and have a high penetrance and severity of arthritis in the absence of any gastrointestinal inflammation (Table 1) [33], [34]. Whereas expression of either transgene alone does not cause disease, expression of both together causes a disease similar to human spondyloarthritis. This Lewis transgenic rat line (21–3×283–2) was chosen for the majority of our studies because 70% develop severe arthritis by 6 months, but the absence of colitis allowed us to avoid the confounding variable of gut inflammation contributing to the changes seen in the gut microbiota. Recent advances in both sequencing technology and our knowledge of the gut microbiome in various mammals including the rat have allowed us for the first time to characterize the differences in the gut microbiota associated with the expression of HLA-B27 and hβ2m.

**Table 1:** HLA-B27/hβ2m transgenic rat lines.

Line	Transgene locus zygosity	Transgene scopies		
		HLA-B27	hβ2m	HLA-B7
21–3	hemi-	20	15	–
	homo-	40	30	–
283–2	hemi-	0	35	–
	homo-	0	70	–
21–3x283–2	hemi- x hemi-	20	50	–
33–3	homo-	55	66	–
120–4	homo-	–	10	52

Adapted from [35].

## Materials and Methods

### Ethics statement

This study was carried out in strict accordance with the recommendations in the Guide for the Care and Use of Laboratory Animals of the National Institutes of Health (NIH). The protocols were approved by the Institutional Animal Care and Use Committee at the University of Texas Southwestern in Dallas, Texas, or the National Institute of Arthritis, Musculoskeletal and Skin Diseases (NIAMS) at the NIH.

### Animals and sample preparation

The transgenic rat lines bred on a Lewis background expressing human HLA-B27 and hβ2m by crossing the 21–3×283–2 lines have been described previously [33]. Male 21–3 rats develop epididymo-orchitis [35]. Male (21–3×283–2)F1 rats develop epididymo-orchitis, peripheral arthritis, and tail spondylitis [33], [35]. None of the males of any of these genotypes develop inflammatory bowel disease [33]. Three main cohorts between 67–70 days old were studied (Table 2): (1) 6 nontransgenic Lewis male rats co-housed with 7 HLA-B27/β2m rats (21–3×283–2 F1 males), and 3 age-matched nontransgenic Lewis males housed separately; (2) 6 nontransgenic Lewis males co-housed with 6 age-matched HLA-B27/hβ2m (21–3×283–3) F1 males; (3) 4 female and 4 male rats of three genotypes: HLA-B27/hβ2m (21–3×283–2 F1), hβ2m (283–2), and nontransgenic, all housed separately by genotype and gender. All of the above rats were produced and housed at the University of Texas Southwestern animal facility under specific pathogen free (spf) conditions. We also investigated a cohort of Lewis rats between 60–180 days old, produced and housed at the NIH in which 28 HLA-B7/ hβ2m rats (line 120–4) were compared to 26 wild type and 28 HLA-B27/hβ2m rats (line 33–3) [36]. All of the 33–3 rats had evidence of colon and cecum inflammation histologically, although the younger animals (8 week old) were clinically normal. The HLA-B7 and wild type animals in this control cohort were phenotypically and histologically normal (Tables 1 and 2).

**Table 2:** Rat cohorts used in experiments.

Cohort	Housing	Gender/Genotype	Samples obtained
1	co-housed	Male/6 WT, 7 (21–3x283–2)F1	whole cecum
	separately housed	Male/3 WT	
2	co-housed	Male/6 WT, 6 (21–3x283–2)F1	cecal lumen, cecal mucosa
3	separately housed by genotype	Male/4 WT, 4 (21–3x283–2)F1, 4 (283–2)	cecal lumen, cecal mucosa
		Female/4 WT, 4 (21–3x283–2)F1, 4 (283–2)	
Control cohort	Depends on age of cohort	Male/15 WT, 15 (33–3), 15 (120–4)	cecal lumen, cecal mucosa
		Female/11 WT, 13 (33–3), 13 (120–4)	

WT: wild type; see Table 1 for genotype designations.

All specimens from cohorts 1–3 were collected at 67–70 days in age, prior to the onset of arthritis. At this age, rats are expected to have an adult repertoire of gut microbiota, but will not develop arthritis for at least another 40 days, so that we could distinguish genotypic effects from phenotypic effects. Whole cecum samples were collected from the first cohort, and cecal luminal contents and cecal mucosal specimens were separately collected from the other two cohorts as well as the control cohort. Following euthanasia, the cecum was removed under sterile conditions and either the intact cecum (including contents) or the cecal mucosa and cecal contents separately were snap frozen in sterile microfuge tubes. Genomic DNA was then extracted from the samples using a Qiagen DNAeasy kit and then sequenced using biome representational in situ karyotyping (BRISK) and/or 16S rRNA gene sequencing techniques (as described below).

### BRISK technique

Phi29 amplification was performed on genomic DNA, and BRISK was performed as previously described [37]. Briefly, genomic DNA was digested with BsaXI to yield 33 bp fragments. Following ligation with ‘barcoded’ Illumina sequencing adapters, these fragments were sequenced at eight samples per lane. Tags are parsed against the reference sequence for rat host tags mapped to the rat genome to provide a karyotype. R^2^ values observed for expected tags per chromosome are calculated, and samples were processed further if R^2^>0.95. Remaining tags were parsed into those associated with known organisms (bacteria, fungi, virus, and parasite). Relative abundance of organisms is then calculated by normalizing the total number of unique tag hits greater than one for that organism by the total number of matched tags or to the total rat host tags present in the specimen when possible. Statistical significance for differences between groups was determined by Monte Carlo analysis, randomly permuting the dataset 100,000 times to establish probabilities for distributions among detected organisms.

### 16S rRNA gene amplification, taxonomic and diversity analysis

A portion of the 16S rRNA gene was amplified using the 515–806 primers as specified by the EMP (http://www.earthmicrobiome.org/) and sequenced on the Illumina MiSeq platform [38]–[42]. Data were quality-filtered using QIIME (Quantitative Insights Into Microbial Ecology) [40]. QIIME was used for alignment using Infernal [43], a stochastic context-free grammar aligner, clustering of sequences into operational taxonomic units using uclust [44], phylogenetic reconstruction using the reference + de novo protocol (given the high incidence of likely new clusters), and taxonomy assignment with the RDP classifier [45]. Alpha and beta diversity analyses and visualizations were also performed [46]–[48]. Statistical significance was assessed using parametric and nonparametric approaches including false discovery rate (FDR) corrections by the Benjamini-Hochberg method.

### Quantitative and semi-quantitative PCR

Confirmation of bacterial species differences between the transgenic and wild type animals was obtained using both traditional PCR and then quantified by real time PCR (RT-PCR) using the ΔΔCt relative quantification method as described previously using the 16S rRNA gene as a reference gene [49]. Primer sequences, annealing temperatures, target genes, and product sizes are listed in Table S1.

## Results

### Expression of the HLA-B27 transgene is associated with alterations in the gut microbiota by 16S rRNA gene sequencing

**Figure 1:**
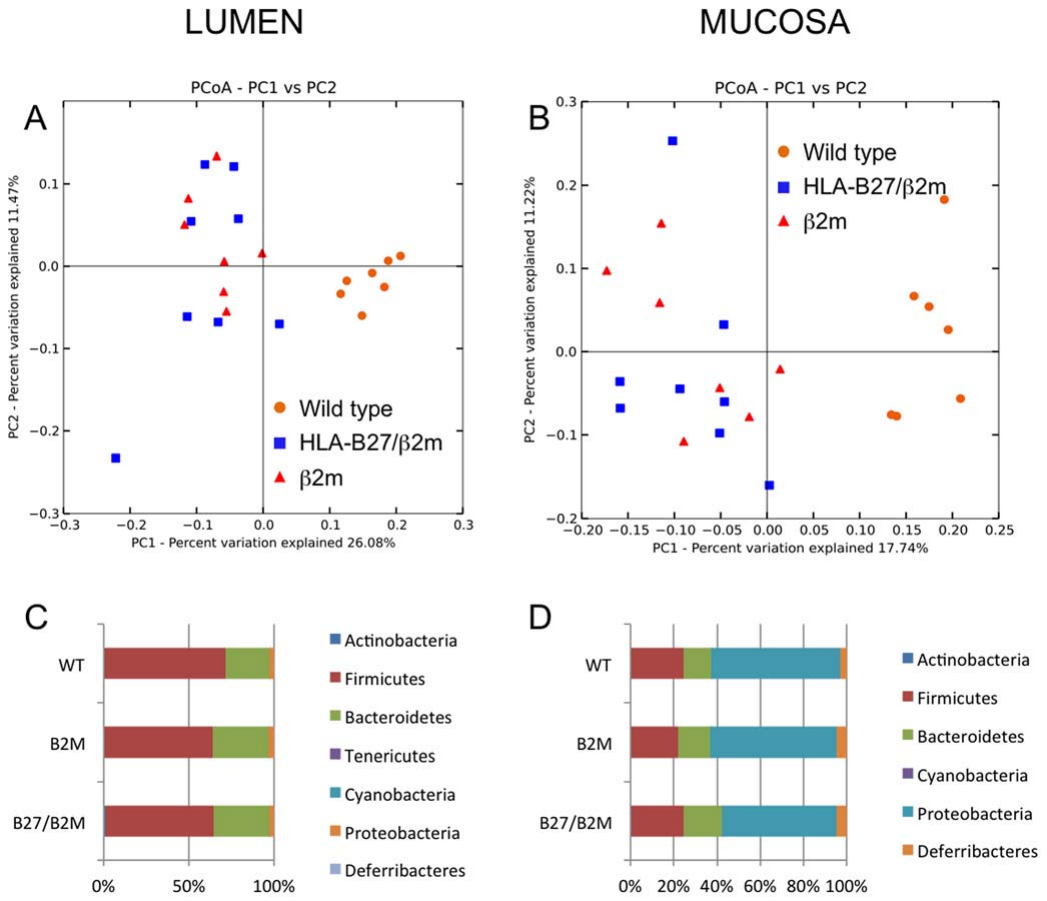
16S rRNA gene sequencing principal coordinates analysis from non-co-housed rats shows significant differences in cecal microbiota between genotypes. A. Cecal lumen samples from cohort 3; B, Cecal mucosa samples from cohort 3; C, Phylum-level analysis from cecum lumen samples; D, Phylum-level analysis from cecum mucosa samples. WT: wild type rats; B2M: hβ2 microglobulin; B27/B2M: HLA-B27/hβ2 microglobulin transgenic rats.

**Figure 2:**
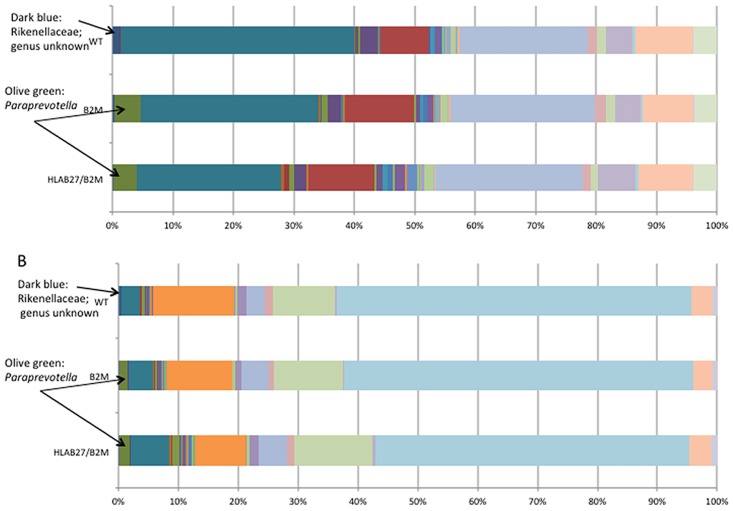
Genus level analysis differences from A, cecal lumen specimens, and B, cecal mucosal samples from cohort 3.

Using 16S rRNA amplicon sequencing, there were statistically significant differences in bacterial sequences between transgenic and wild-type animals in both cecal lumen and cecal mucosa samples shown by principal coordinates analysis using UniFrac distances [50] (Figure 1a, 1b), with p-values shown in Table 3 by PERMANOVA analysis. While phylum-level differences were not statistically significant (Figure 1c, 1d), genus-level differences between genotypes were more easily distinguishable (Figure 2). Whereas the more abundant bacterial genera (such as *Clostridia* and *Helicobacter*) did not differ between transgenic and wild type animals, some of the rarer genera differed markedly. In both cecal lumen and cecal mucosa samples, an unknown genus of the Rikenellaceae family (dark blue bars) was significantly more abundant in wild type animals than either HLA-B27/hβ2m or hβ2m animals (p = 0.0005 and 0.002, respectively after FDR correction). On the other hand, *Paraprevotella* was higher in transgenic (HLA-B27/β2m or β2m) animals compared to the wild type animals (p = 0.03 in both tissue sites after FDR correction).

**Table 3:** HLA-B27 transgenic animals have significant alterations in gut microbiota by 16s rDNA sequencing.

	Animal group comparison	Permanova analysis, p value	n from each group
Co-housed animals	Whole cecum (WT vs B27/B2M)*	1.9066, 0.006	6 vs 7
	Cecal mucosa (WT vs B27/B2M)	1.2121, 0.1688	6 vs 6
	Cecal lumen (WT vs B27/B2M)	1.0113, 0.4266	6 vs 6
Non-co-housed animals	Cecal lumen (WT vs B27/B2M vs B2M)*	3.4031, 0.001	7 vs 8 vs 7
	Cecal lumen (WT vs B27/B2M)*	4.7903, 0.001	7 vs 8
	Cecal lumen (WT vs B2M)*	5.1028, 0.001	7 vs 7
	Cecal lumen (B27/B2M vs B2M)	0.76438, 0.7652	8 vs 7
	Cecal mucosa (WT vs B27/B2M vs B2M)*	2.4418, 0.001	7 vs 8 vs 7
	Cecal mucosa (WT vs B27/B2M)*	3.1632, 0.002	7 vs 8
	Cecal mucosa (WT vs B2M)*	3.0912, 0.001	7 vs 7
	Cecal mucosa (B27/B2M vs B2M)	1.0951, 0.3017	8 vs 7

WT: wild-type Lewis rats; B27/B2M: (21–3×283–2)F1 HLA-B27/hβ2m transgenic Lewis rats; B2M: 283–2 hβ2m expressing transgenic rats; * designates p<0.05.

**Figure 3:**
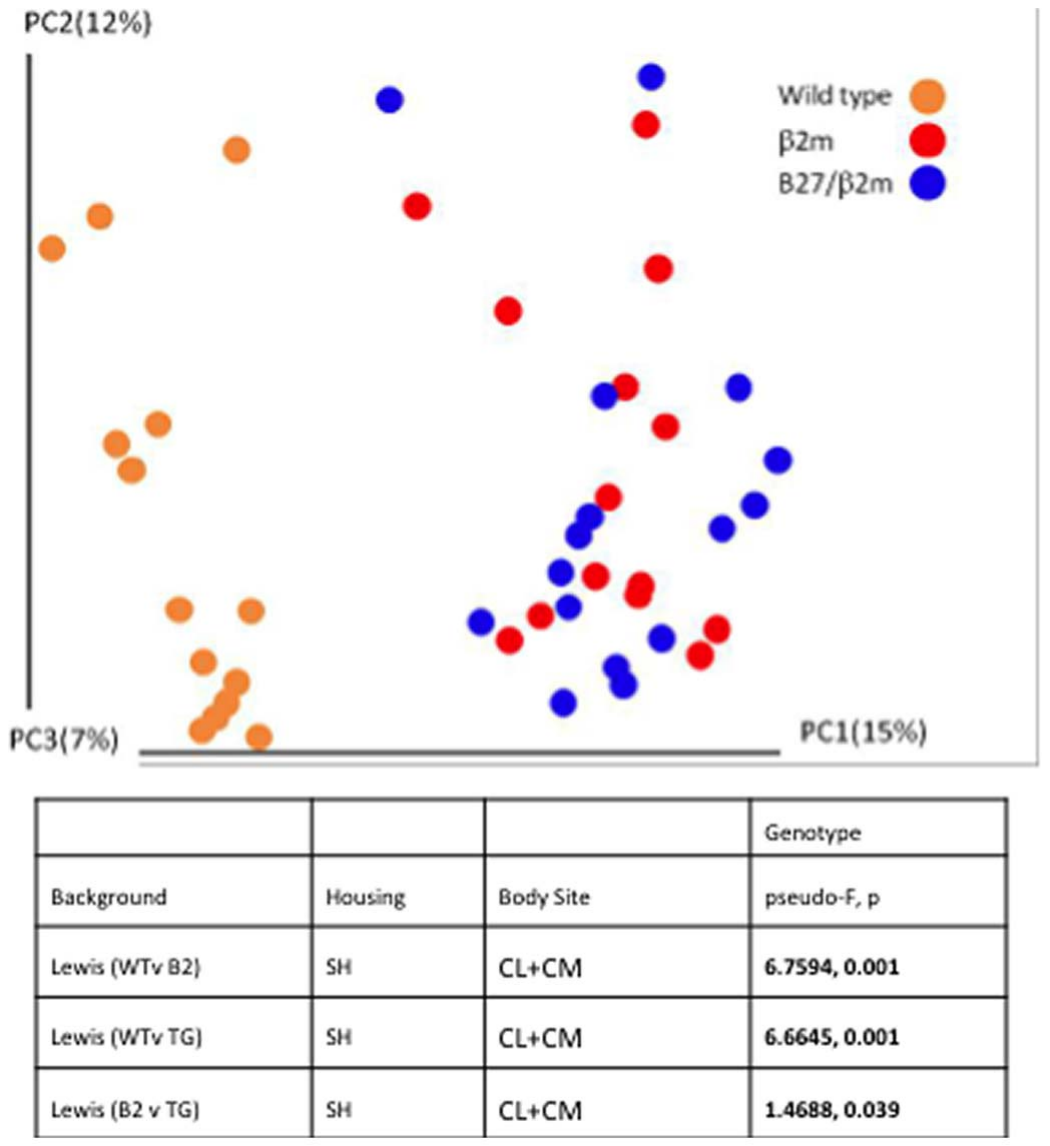
Principal coordinates analysis from combined cecal lumen and cecal mucosa samples from cohort 3.

In the 3^rd^ cohort of animals, whereas the represented bacterial sequences obtained from the HLA-B27/hβ2m animals from combined cecal lumen and mucosa samples segregated significantly from the wild type animals (pseudo-F 6.6645, p = 0.001) on principal coordinates analysis, there was a much smaller difference between the HLA-B27/hβ2m rats and rats that only expressed only hβ2m (pseudo-F 1.4688, p = 0.039) (Figure 3). Table 3 also illustrates that co-housing abrogated some of the differences seen between transgenic animals and wild type animals (cohort 2). This might be expected because rodents are coprophagic. However, in whole cecum preparations from the first cohort of animals, there remained significant differences in the Unifrac distances between the gut microbiota of wild type and HLA-B27/hβ2m transgenic animals despite co-housing (p = 0.006) (Table 3). HLA-B7, like HLA-B27, is also an MHC I allele, but differs in that it is not associated with arthritis susceptibility and thus serves as an MHC class I control. Significant differences in the gut microbiota were demonstrated in HLA-B7/ hβ2m compared with both wild type controls and HLA-B27/hβ2m rats (pseudo-F 4.6750, p = 0.001 by permanovafor cecal lumen and pseudo-F 5.6001, p = 0.001 by permanova for cecal mucosa). Figure S1 demonstrates the separation of 16s sequences by genotype using principle coordinates analysis and table S2 shows the p values for genotype group comparisons in this control cohort.

### Expression of the HLA-B27 transgene is associated with bacterial species-level alterations in the gut microbiota by BRISK

**Figure 4:**
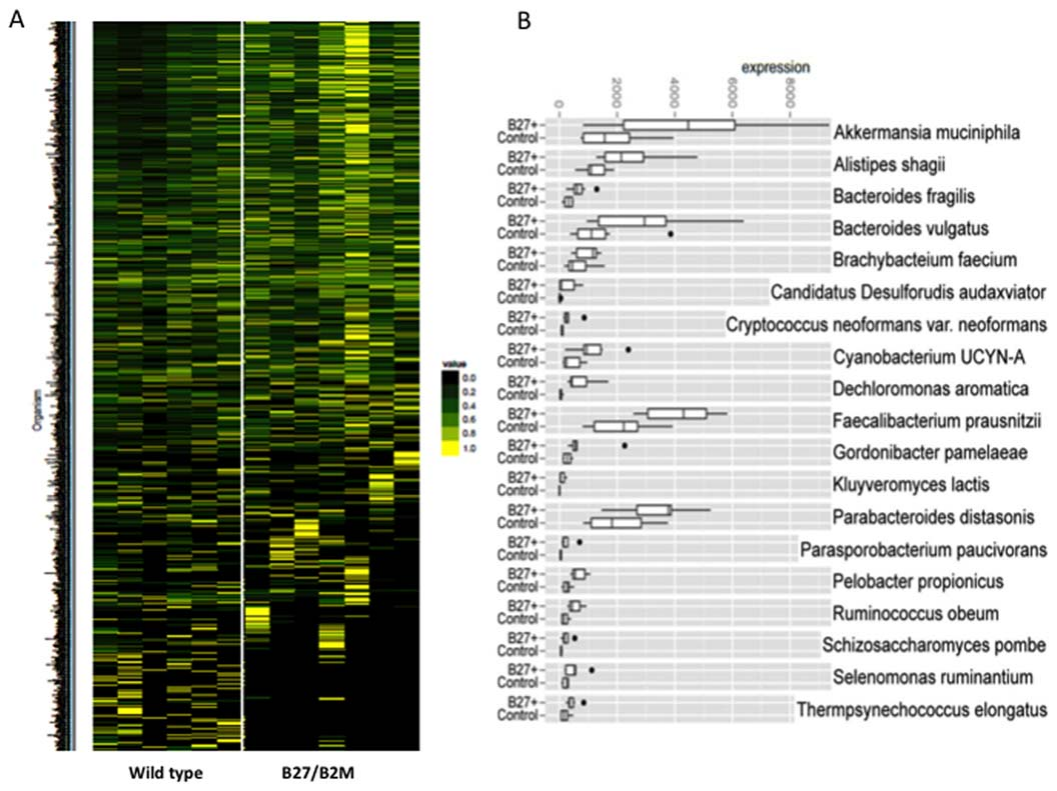
BRISK analysis of whole cecum samples from cohort 1 shows microbial diversity differences on a species level between HLA-B27/hβ2m animals and co-housed wild type animals. A. Each line on the heat map represents a different bacterial species and the color code represents the relative abundance of that organism, with yellow indicating the highest abundance. B, Specific species differences are highlighted, with a black dot indicating statistically significant differences.

BRISK is a technique that allows for massively parallel deep sequencing of the gut microbiome generated by a Type IIB restriction endonuclease. This provides a representation of the specific microorganisms present in a sample on a species level, as well as the abundance of these organisms. In a typical sample, we were able to amplify about 4 million tag, with ∼21% of the DNA identified as from the host animal and representing all chromosomes inthe rat genome. Across all samples we identified more than 30,000 tags derived from known bacteria. In total, tags from 909 species were identified in at least one animal. The heat map (Figure 4) shows the relative abundance of specific bacterial species from whole cecum preparations of transgenic rats compared to wild type control animals from the first cohort (normalized to amount of host rat DNA), demonstrating an overall difference in the abundance of certain bacterial species. The color code represents the relative abundance of specific bacterial species with each row showing an individual species. Figure 4b quantifies differences between HLA-B27/hβ2m transgenic animals and littermate wild type control animals (co-housed animals). Approximately 30 of these showed significant differences in HLA-B27/β2m transgenic animals compared to wild type animals as determined by iterative Monte Carlo analysis of the dataset. Three of the main differences included alterations in the abundance of *Faecalibacterium prausnitzii*, *Bacteroides vulgatus*, and *Akkermansia muciniphila*.

### *Bacteroides vulgatus* is more abundant in HLA-B27 transgenic animals than non-co-housed wild type animals

**Figure 5:**
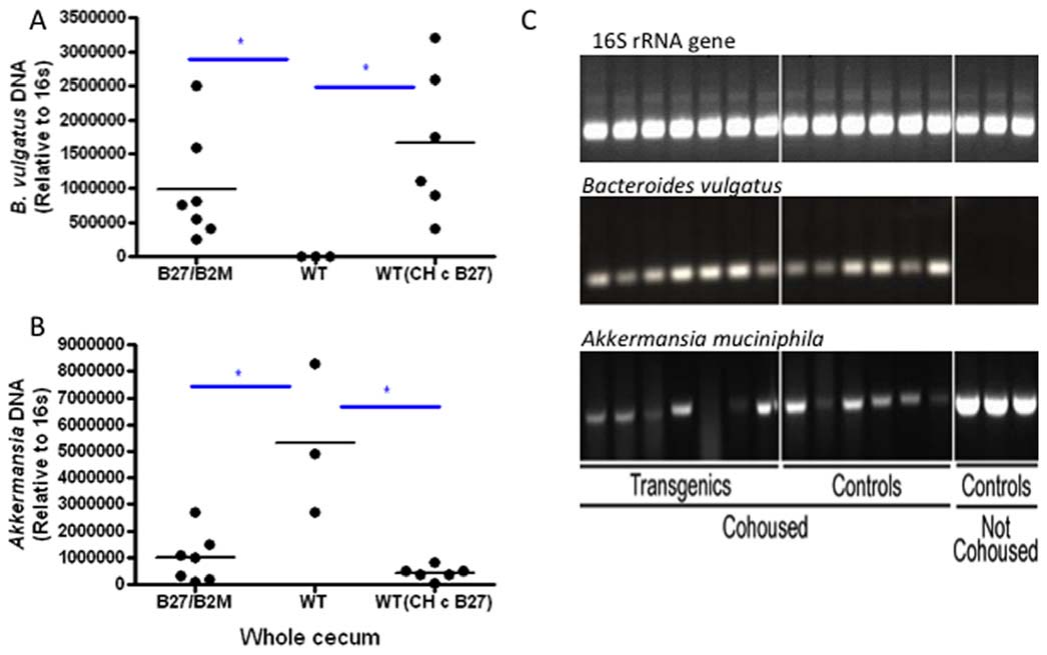
Relative differences in *Bacteroides vulgatus* and *Akkermansia muciniphila* shown by A, quantitative PCR using the 16S rRNA gene as a reference, and B, by traditional PCR using whole cecum samples in cohort 1.

We next attempted to confirm HLA-B27-dependent differences in colonization of these three bacterial species using species-specific real time quantitative PCR. Normalization was performed using 16S rRNA gene PCR. The differences in *F. prausnitzii* could not be confirmed by qPCR. However, we found a marked reduction in *Akkermansia muciniphila* and an increase in *Bacteroides vulgatus* prevalence in the transgenic HLA-B27/hβ2m animals compared to the wild type rats housed alone (Figure 5). However, the differences were reduced (with levels of these bacteria in wild-type mice comparable to transgenic) when wild type animals were co-housed with the transgenic animals, presumably due to coprophagia. Although there were significant differences in *Bacteroides vulgatus* by BRISK in both co-housed and non-co-housed animals, when confirmatory PCR was performed, a difference was only demonstrated between non-co-housed animals. In cohort 3, *Akkermansia muciniphila* was not present in co-housed and non-co-housed rat cecal samples (neither cecal mucosa nor cecal lumen), suggesting that this species may have colonized this specific group of wild type animals only.

## Discussion

To our knowledge this is the first study that shows that expression of the human transgene HLA-B27 (with hβ2m) is associated with alterations in the gut microbiota. The purpose of this study was to establish the difference seen in the gut microbiota due to HLA-B27, but whether or not specific changes are causative of disease, a result of disease, or unrelated to disease has not yet been established and will require further investigations. Germ-free studies in the 33–3 and 21–4 lines strongly suggest that gut microbiota play an important role in the disease state in this animal model [21]–[23]. The effect of the germ free state on arthritis in the (21–3×283–2) F1 rats which do not develop colitis remains to be studied.

The rat age in our study (in the first 3 cohorts) was specifically chosen to precede arthritis onset so that the genotype effect could be delineated from the disease effect. At 67–70 days, (21–3×283–2)F1 males show only very localized inflammation in the male genital tract, but no gastrointestinal inflammation or joint inflammation [35]. It is possible that HLA-B27 animals have a distinct T cell selection repertoire resulting in bacterial peptide presentation in a way that affects which microbes are allowed to thrive and/or are eliminated. However, studies that cross a CD8 −/− background to the Lewis 33–3 rat have shown no effect on disease prevalence or severity, thus diminishing the validity of this theory [51]. While there is no evidence that the (21–3×283–2)F1animals used in this study have gut inflammation both histologically and by lack of increased cytokine production, the 33–3 rats do have subclinical inflammation from an early age (8 weeks old). Sartor and colleagues have shown that gut inflammation in the Fischer HLA-B27 33–3 transgenic rats which develop clinically-evident inflammatory colitis alters the transcriptional profile of *Bacteroides thetaiotamicron,* which is thought potentially to be a causative organism in colitis, and may result in an augmented adaptive immune response to bacterial antigen [52]. Analogously to the human disease, ankylosing spondylitis, in which 50–60% of patients without GI symptoms have microscopic gut inflammation on histological analyses of biopsy specimens from routine colonoscopy, it is possible that transgenic rats have subclinical gut inflammation that causes changes in the gut microbiota, or alternatively, that transgene expression directly changes the members of the gut microbiota which results in alterations in immunity and/or gut permeability.

We have demonstrated by species-specific sequencing and qPCR confirmation that *Bacteroides vulgatus* is overabundant in the HLA-B27 rat ceca. This organism was found in mono-association studies in germ-free HLA-B27 transgenic Fischer rats to induce colitis [23], [53]. While the Lewis transgenic rats used in the first 3 cohorts of our study do not develop colitis, it is possible that *B. vulgatus* is pathogenic in the development of arthritis. Testing this hypothesis would require longitudinal correlation of the presence of *B. vulgatus* with arthritic disease score severity, and/or monoassociation of germ-free rats with *B. vulgatus* to induce arthritis. It is also possible that the overabundance of *B. vulgatus* only reflects more global changes in the microbiota and is not directly related to disease.

We also found that *Paraprevotella* was more abundant in both transgenic animals (HLA-B27/β2m and β2m) by 16s rRNA gene sequencing compared with wild type animals. *Prevotella* species, which are related to *Paraprevotella* have been implicated in the pathogenesis of rheumatoid arthritis in humans [54].

While the components of gut microbiota in different groups of animals can differ depending on the specific micro-environment, as shown by the failure to confirm loss of abundance of *Akkermansia* in our repeat experiments, an overall consistent trend was observed that transgenic animals had different microbiota compared to wild type animals, even in the absence of gut inflammation. Given that individual bacterial species might be inconsistent between groups of transgenic animals, the HLA-B27 disease association might rather be due to broader differences in the metabolic profiles of the bacterial species present, and thus, it is possible that disease and altered immunity may result from the overall milieu of bacterial metabolites interacting with the host rather than an alteration of individual bacterial species.

The transgenic animals that only expressed hβ2m without HLA-B27 exhibited microbiota differences that were more similar to HLA-B27-expressing animals than wild type animals despite being separately housed, although there were some microbiota differences unique to HLA-B27. The hβ2m transgenic animals remain completely healthy in this cohort, while all of HLA-B27/hβ2m rats develop epidiymoorchitis and a majority develop arthritis. One potential explanation for these results could be that the changes in microbiota are at least in part due to the expression of hβ2m rather than HLA-B27, but these alterations are not pathogenic unless HLA-B27 is also present, causing disease either by effecting additional changes in the microbiome, and/or by contributing its effect(s) on the immune response. Another potential explanation is that human β2m may form a functional complex with rat MHC class I heavy chains, provoking a change in the microbiota that by itself is insufficient to cause disease. There is evidence that overexpression of human HLA-DR*0401 and* 0402, HLA-DR4 subtypes that cause mice to be susceptible or resistant to arthritis, respectively, results in genotype-specific alterations in the gut microbiota [55]. We also show in this study that another MHC I molecule, HLA-B7, can be associated with changes in the gut microbiota differentially from both wild type and HLA-B27 animals. Overall, our results support the concept that changes in MHC expression influence the composition of the gut microbiota. Effects of HLA-B27 that cause disease could turn out to be an unusual specific case within this general principle.

One potential limitation to our study is that random drift, as well as seasonal changes, can contribute to differences detected in the gut microbiome. However, because we compared the microbiota between genotypic groups harvested at the same time, this should not be a large factor in the outcome of our analyses. For instance, within cohort 1, we harvested all genotypes at the same time (as within cohort 2 and 3). Since we have not attempted to pool the three cohorts (which were indeed euthanized at different times) for analyses, random drift should be much less of a factor. It should also be noted that B27+/hβ2m+ rats were littermates of B27-/ hβ2m+ rats in that particular cohort (cohort 3), so these two groups should have the same founder microbiome and differences seen between these two genotypes would argue against drift being the main factor. In terms of differences due to different founder microbiomes, this is theoretically a problem with the wild type rats even though husbandry was similar for all rats at a particular time. While this does not necessarily mitigate the founder effect, we have at least demonstrated that in co-housed rats some genotypic differences exist.

Sequencing techniques have advanced significantly in the last decade to allow for more rapid and in-depth methods of identifying, in a culture-independent fashion, the members of a complex microbial community such as the gut. It is important to note that there are strengths and limitations to the two methods used here. For instance, while the 16s rRNA gene sequencing method can provide better diversity comparisons between two groups in a cohort using principal coordinates analysis, detailed species-specific information was not readily available with the taxa we used in our analysis.It is sensitive to dramatic changes in gut microbiota such as occur with overt clinical gut inflammation, but may fail to find identify potentially crucial differences on a species level. On the other hand, while the BRISK technique is able to identify species-level differences, its sensitivity is limited by identification of specific tags within the database. While 16S sequences are known for many bacteria, BRiSK can generally only identify organisms for which full genome sequences are available. Potentially pathogenic organisms may be among the species that have not been identified or described. Despite these limitations, we have identified substantial microbial biodiversity differences in the gut between wild type and transgenic rats. These findings can potentially direct us to better understand immune-mediated diseases associated with HLA-B27.

## Supporting Information

Figure S1 **16S rRNA gene sequencing principal coordinates analysis demonstrates significant differences between HLA type in Lewis rats between 2–6 months of age from A, cecal lumen and B, cecal mucosa.** B27/β2M: HLA-B27/human β2-microglobulin; B7/β2M: HLA-B7/human β2-microglobulin. Cecal mucosal and lumenal contents from rats in various cohorts were collected using sterile swabs and frozen in sterile 15 ml centrifuge tubes. Samples were shipped to OHSU in dry ice, where genomic DNA was extracted using Qiagen DNAeasy kit and sequenced using 16S rRNA gene sequencing. Control cohorts were handled as follows: 2 month old rats: Weaned at 21 days after birth and singly housed until 2 months of age and sacrificed. 3–4 month old rats: Weaned at 21 days and cohoused with litter mates (random transgenic and wild type) usually 2–3 rats per cage until almost 3 months of age when they were sacrificed. 6 month old rats: Weaned at 21 days and cohoused with litter mates (random transgenic and WT) usually 2–3 rats per cage until almost 3 months of age and then singly housed. Some of these animals were cohoused for mating for a few weeks intermittently.
(TIF)

Table S1 **Primers used for quantitative PCR.**
(DOCX)

Table S2 **Bonferroni corrected p values corresponding to Figure S1 demonstrating differences seen in control cohort Lewis rats including HLA-B7 transgenic rats.**
(DOCX)
