# Supplementary figures and images for: HLA-B27 and Human β2-Microglobulin Affect the Gut Microbiota of Transgenic Rats
Source: PLoS One. 2014 Aug 20;9(8):e105684. doi: 10.1371/journal.pone.0105684 (PMC4139385; doi:10.1371/journal.pone.0105684)

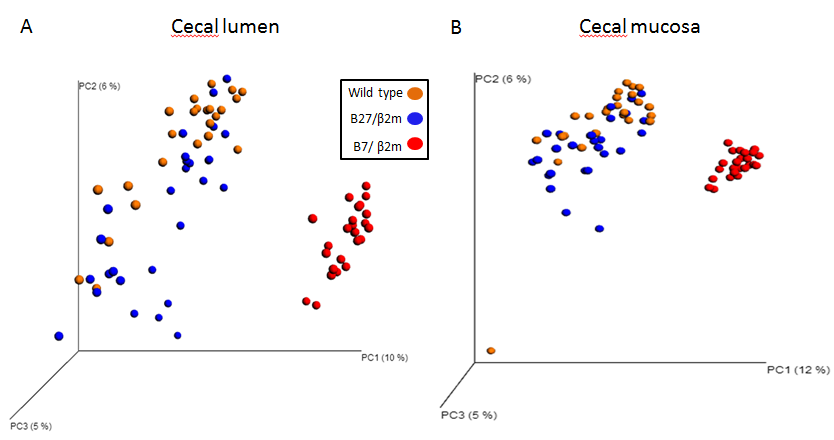

Supplement: Figure S1 — 16S rRNA gene sequencing principal coordinates analysis demonstrates significant differences between HLA type in Lewis rats between 2–6 months of age from A, cecal lumen and B, cecal mucosa. B27/β2M: HLA-B27/human β2-microglobulin; B7/β2M: HLA-B7/human β2-microglobulin. Cecal mucosal and lumenal contents from rats in various cohorts were collected using sterile swabs and frozen in sterile 15 ml centrifuge tubes. Samples were shipped to OHSU in dry ice, where genomic DNA was extracted using Qiagen DNAeasy kit and sequenced using 16S rRNA gene sequencing. Control cohorts were handled as follows: 2 month old rats: Weaned at 21 days after birth and singly housed until 2 months of age and sacrificed. 3–4 month old rats: Weaned at 21 days and cohoused with litter mates (random transgenic and wild type) usually 2–3 rats per cage until almost 3 months of age when they were sacrificed. 6 month old rats: Weaned at 21 days and cohoused with litter mates (random transgenic and WT) usually 2–3 rats per cage until almost 3 months of age and then singly housed. Some of these animals were cohoused for mating for a few weeks intermittently. (TIF) [file pone.0105684.s001.tif]
