# Supplementary material for: HLA-B27 and Human β2-Microglobulin Affect the Gut Microbiota of Transgenic Rats
Source: PLoS One. 2014 Aug 20;9(8):e105684. doi: 10.1371/journal.pone.0105684 (PMC4139385; doi:10.1371/journal.pone.0105684)
Supplement: Table S1 — Primers used for quantitative PCR. (DOCX) [file pone.0105684.s002.docx]

**Table S1**. Primers used for quantitative PCR.

| Primer name | Forward primer | Reverse primer | Amplicon size (bp) |
| --- | --- | --- | --- |
| 16S | 5’CTGGAGCGCTATGCTGTACGATG3’ | 5’AGGCCCGGGAACGTATTCAC3’ | 236 |
| *Akkermansia muciniphila* | 5’TGCCCAATGCCATTACCCTGAC3’ | 5’GTGCAGGCAGCCAGAATGAAC3’ | 127 |
| *Bacteroides vulgatus* | 5’CTCTTCTGTCTGCGTGATGATTTC3’ | 5’CCTGCTGCTGGTGATTGCTATCC3’ | 223 |
| *Faecobacterium prausnitzii* | 5’TCGGACACGGTCATCATGTTGTAGTC3’ | 5’CTGGAGCGCTATGCTGTACGATG3’ | 225 |

bp: base pairs
