# Supplementary material for: HLA-B27 and Human β2-Microglobulin Affect the Gut Microbiota of Transgenic Rats
Source: PLoS One. 2014 Aug 20;9(8):e105684. doi: 10.1371/journal.pone.0105684 (PMC4139385; doi:10.1371/journal.pone.0105684)
Supplement: Table S2 — Bonferroni corrected p values corresponding to Figure S1 demonstrating differences seen in control cohort Lewis rats including HLA-B7 transgenic rats. (DOCX) [file pone.0105684.s003.docx]

**Table S2.** Bonferroni corrected p values corresponding to supplemental figure 1 demonstrating differences seen in control cohort Lewis rats including HLA-B7 transgenic rats.

| Tissue site | WT vs B7 | B7 vs B27 | WT vs B27 |
| --- | --- | --- | --- |
| Cecal lumen | 5 x 10^-37^ | 2 x 10^-95^ | 6 x 10^-20^ |
| Cecal mucosa | 5 x 10^-17^ | 2 x 10^-42^ | 7.91 x 10^-24^ |

WT: wild type rats; B7: HLA-B7/hβ2m transgenic rats; B27: HLA-B27/ hβ2m transgenic rats
